# The Low Mini-Midvastus Approach for Minimally Invasive Total Knee Arthroplasty

**DOI:** 10.1016/j.artd.2025.101686

**Published:** 2025-04-24

**Authors:** Kein Boon Poon, Justin Zi Xian Chou, Zachariah Gene Wing Ow, Joel Wei-An Lim, Wei Ming Siow

**Affiliations:** Department of Orthopaedic Surgery, Sengkang General Hospital, Singapore

**Keywords:** Total knee arthroplasty, Minimally invasive surgery, Knee osteoarthritis, Surgical techniques

## Abstract

The low mini-midvastus approach is a refinement of minimally invasive techniques in total knee arthroplasty (TKA). This technique involves making a precise muscle-splitting incision along the vastus medialis obliquus, retaining a 1-cm wide inferomedial cuff of the muscle belly, preserving the extensor mechanism while maintaining sufficient surgical exposure. We hypothesize that the low mini-midvastus approach minimizes postoperative pain, accelerates recovery, and enhances patient satisfaction by optimizing vastus medialis obliquus integrity. It is suitable for primary TKA and adaptable to challenging anatomical variations. In this study, we describe our surgical technique and experience with such an approach for conventional primary TKA that represents a refinement of the midvastus spectrum that prioritizes muscle preservation while maintaining adequate exposure.

## Introduction

The evolution of minimally invasive surgical (MIS) techniques in total knee arthroplasty (TKA) reflects a progressive commitment to improving patient outcomes. These approaches aim to minimize disruption to the quadriceps extensor mechanism while maintaining reliable and reproducible knee joint exposure. Additionally, they facilitate the introduction of well-sized prostheses, with the overarching goals of improving patient satisfaction and accelerating postoperative recovery. Traditional TKA procedures often involved extensive incisions and significant disruption of soft tissue, particularly the quadriceps mechanism, which delayed recovery and increased postoperative pain. Over the past 2 decades, MIS approaches have revolutionized this field, focusing on muscle preservation, specifically the vastus medialis obliquus (VMO) and reducing the invasiveness of surgery, thereby accelerating early to mid-term functional recovery [1,2].

Among the widely accepted MIS techniques, the midvastus (MV) and mini-midvastus (mMV) approaches have gained prominence due to their ability to preserve the integrity of the quadriceps tendon while offering sufficient exposure of the knee joint. The mMV approach, in particular, has been hailed as a technique that achieves a delicate balance between minimal quadriceps musculature disruption and adequate surgical access [3]. However, while this technique reduces muscle trauma compared to the medial parapatellar approach, there is still disruption to the VMO that could be further minimized. Furthermore, by involving the small degree of VMO splitting, surgical exposure of the knee joint is improved, resulting in shorter tourniquet and operative times compared to muscle-sparing approaches such as the subvastus (SV) and quadriceps sparing techniques [4].

Despite these advantages, the mMV approach still involves sacrificing a segment of the VMO muscle, which raises questions about whether this sacrifice could be further minimized without compromising surgical outcomes and preserving adequacy of exposure.

Thus, we introduce our modification of the mMV technique, the low mini-midvastus (low-mMV) approach, which preserves a larger proportion of the VMO muscle bulk by limiting the split to a 1-cm cuff along the inferomedial margin. Other parameters of our approach are in keeping with established definitions of the mMV approach, such as a skin incision <12 cm and VMO split extension of <3 cm from the patellar margin [5,6].

The low-mMV approach is an extension of the mMV spectrum rather than a new paradigm shift, refining the technique for improved muscle preservation. While some aspects resemble the SV approach, our technique retains the advantages of the MV approach by allowing greater flexibility in cases with challenging anatomy. Through this article, we seek to establish the low-mMV approach as a progressive step forward in minimally invasive techniques for TKA.

## Surgical technique

### Patient evaluation, imaging, and indications

Our technique of a low-mMV approach is used as the default choice of incision for all primary conventional TKAs. Our technique is performed in a single institution by a single surgeon using the conventional nonrobotic-assisted approach for TKAs. All patients set to undergo a TKA are evaluated preoperatively with weight-bearing radiographs of the affected knees in orthogonal planes, as well as a skyline view for patellofemoral evaluation, and a long-limb radiograph to evaluate native lower limb alignment.

One relative contraindication to using our low-mMV incision would be that of revision TKA, as well as morbid (class III) obesity defined as a body mass index of more than 35 [7] owning to the requirement for more extensive exposure to the knee joint in such patients. Factors such as severe varus or valgus deformities, chronic patellar dislocations requiring extensive lateral releases, use of robotic navigation, well-developed quadricep musculature, or preoperative flexion deformities were not strict contraindications to employing our low-mMV approach; however, the total length of VMO dissection was occasionally extended by 2-3 cm in such cases wherein exposure was prohibiting progression of the surgery.

### Patient positioning and anesthesia

The patient is positioned supine, and the surgery is carried out under either general anesthesia with an adductor block or spinal anesthesia. Footrests are positioned to allow for the knee to be held in 90° as well as hyperflexion, and a side support is placed to keep the leg aligned parallel to the sagittal axis. A high thigh tourniquet is placed and inflated at the commencement of skin incision, and first-generation cephalosporins are administered as routine perioperative antibiotics unless otherwise contraindicated.

### Skin incision and deep dissection

**Figure 1:**
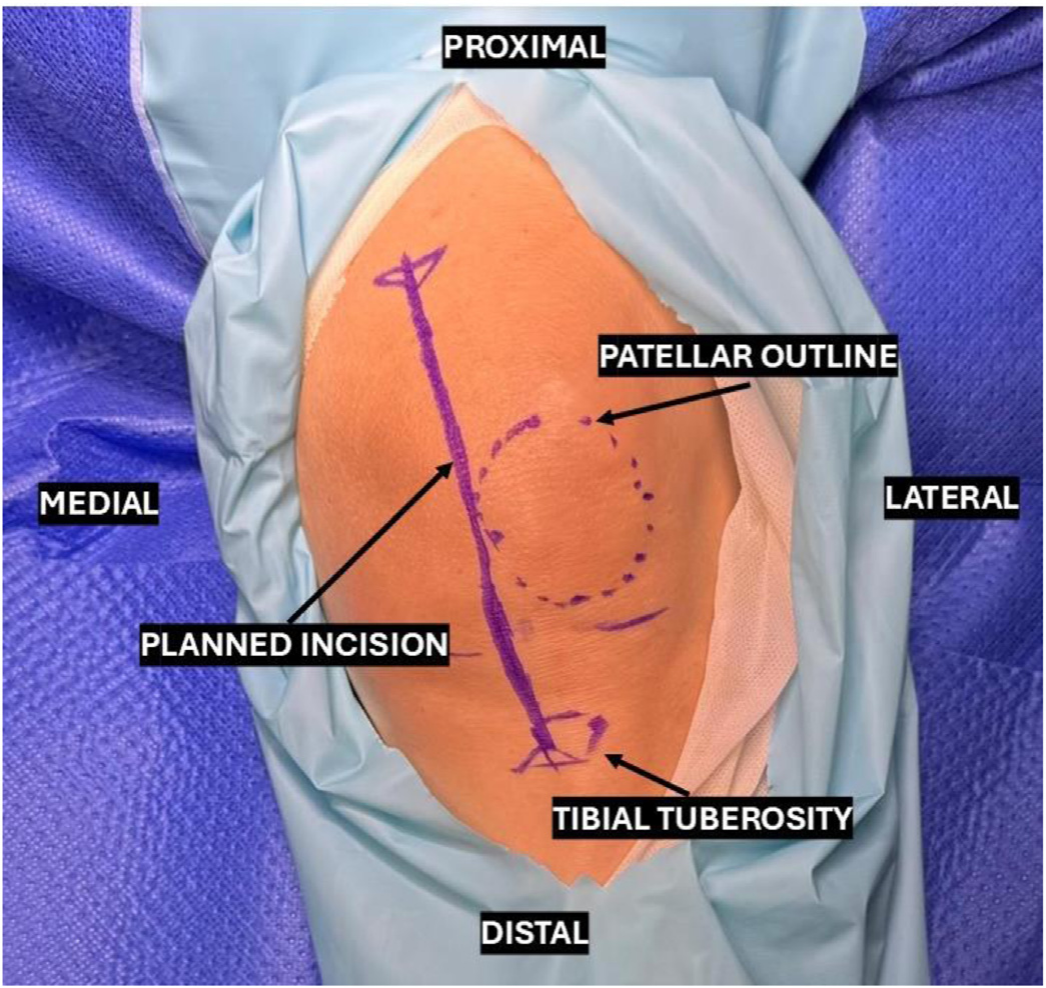
Templating the skin incision.

With the knee held in 90° flexion, the landmarks for our skin incision are identified. The superior, inferior, medial, and lateral borders of the patella are identified, and a straight incision is templated with a surgical skin marker, originating 2 fingerbreadths proximal to the superior pole of the patella, and extending slightly obliquely across the medial border of the patella in the direction of the tibial tuberosity, and ending 2 fingerbreadths distal to the inferior margin of the patella. The length of this skin incision is reliably less than 12 cm when templated in the above manner (Figure 1).

**Figure 2:**
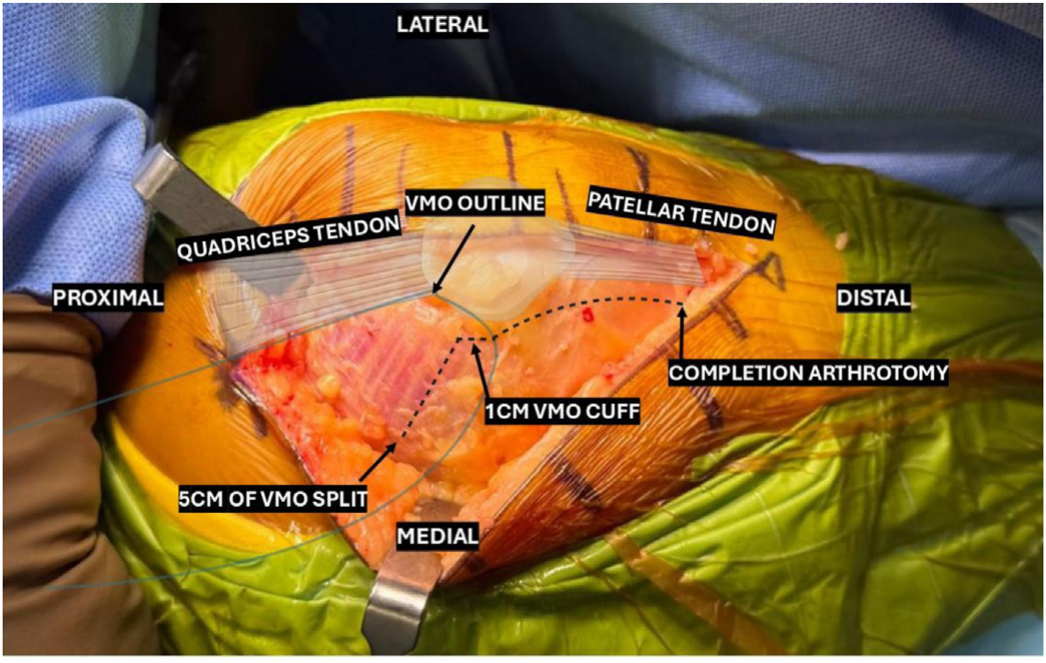
Illustration of the low-mMV incision in relation to anatomical landmarks, pictured is a left knee photographed from a medial viewpoint. low-mMV, low mini-midvastus.

Superficial dissection continues through the planes of loose connective tissue until the depth of VMO epimysium is reached. At this stage, the VMO, medial parapatellar retinaculum, and patella are clearly visible (Figure 2).

**Figure 3:**
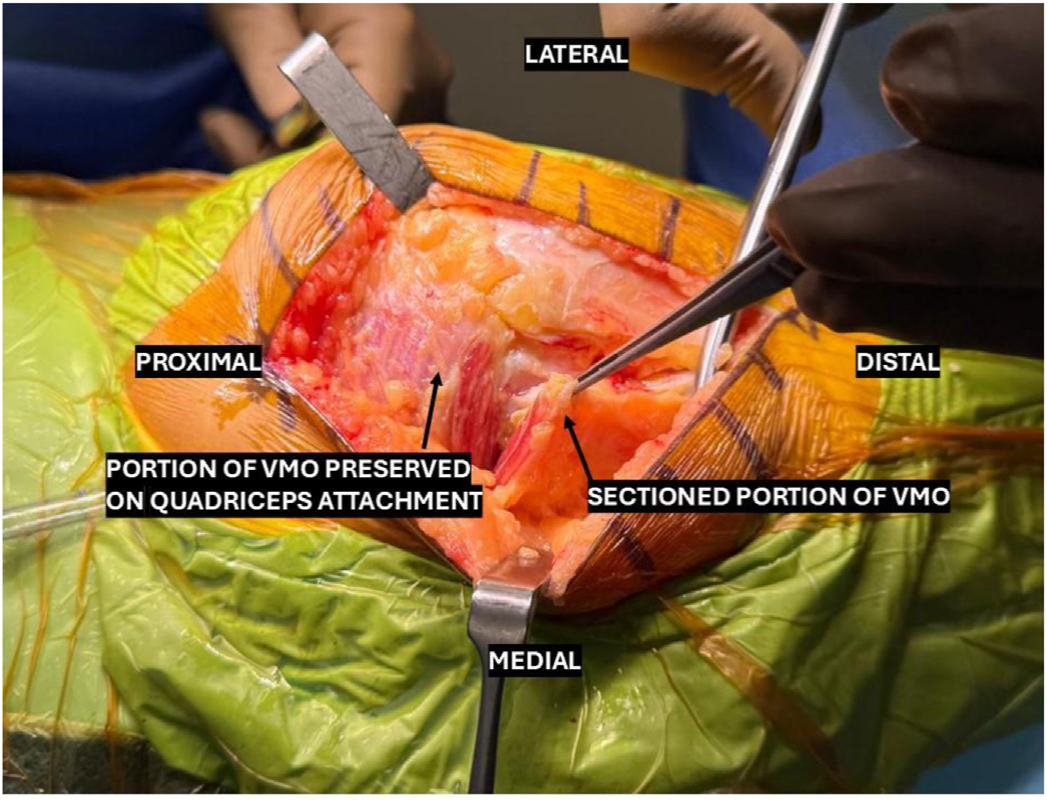
The completed low-mMV VMO split showing minimal sectioning of the muscle bulk. low-mMV, low mini-midvastus; VMO, vastus medialis obliquus.

The deep incision is then initiated by incising vertically through the medial parapatellar retinaculum at the level of and distance of 5 mm to the medial patellar facet. This incision is then extended proximally, sectioning an inferomedial cuff of VMO fibers up to a width of 1 cm. The incision is then acutely angled superiorly and medially, splitting the VMO fibers along the direction of travel, and extending no further than 5 cm proximal to the patellar margin. Focus is then diverted to completing the distal deep incision by dividing the knee capsule vertically until the level of the patellar tendon attachment to the tibial tuberosity (Figure 3).

**Figure 4:**
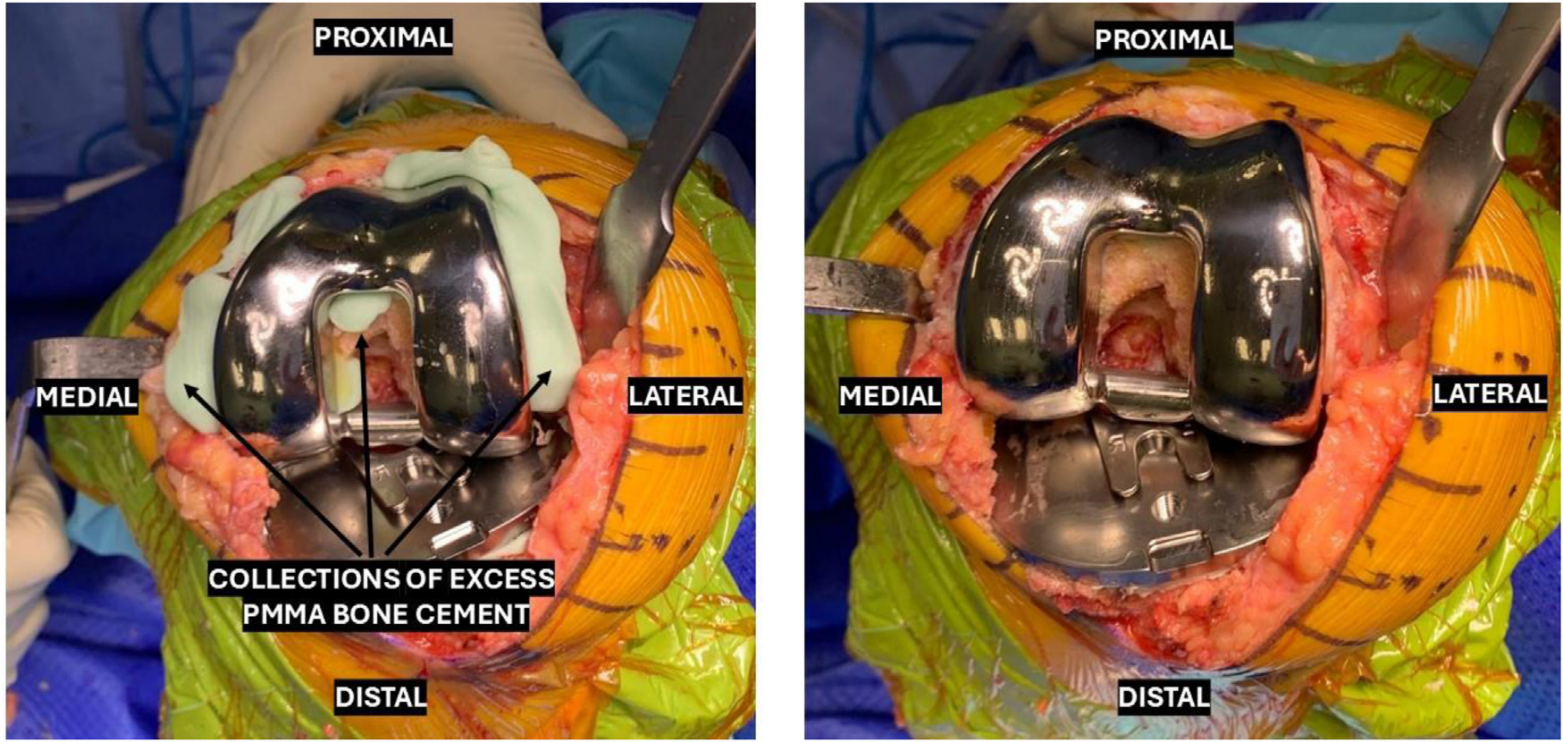
Exposure of the implants and clearing excess polymethylmethacrylate (PMMA) bone cement.

Once the arthrotomy is completed, the knee is extended to allow for patellar fat pad excision, prior to being repositioned to hyperflexion, wherein the patella is laterally subluxed, capsular and medial releases are performed, and the anterior cruciate ligament and menisci are excised. Exposure is maintained using a Z-retractor placed medially deep to the medial collateral ligament and a straight bladed Homann retractor placed laterally to keep the patella in eversion. The rest of the procedure is continued in routine fashion in accordance with the technical instructions of the respective implant system. All implants are cemented due to individual surgeon preference, and the choice of posterior stabilized or cruciate-retaining constructs is determined based on patient and surgical factors. Despite the minimally invasive approach, our technique allows for adequate exposure in inserting implants and clearing access polymethylmethacrylate bone cement (Figure 4).

Upon completion of implant insertion, the joint is irrigated, and closure is performed in a layered fashion, with special care taken to ensure the knee joint capsule is repaired with a watertight seal. The skin incision is closed with a barbed dissolvable suture in a subcuticular fashion.

### Postoperative protocol

Following a postoperative assessment by the physiotherapy team, patients are ambulated with full weight-bearing on the operated leg starting from 4 hours after surgery, with aggressive bed exercises in between ambulation sessions. Basal analgesic coverage is maintained solely with oral agents comprising paracetamol, nonsteroidal anti-inflammatory agents, and weak opiates such as tramadol.

## Discussion

The low-mMV approach for minimally invasive TKA represents a refinement rather than a reinvention of the MV technique. The foundational goal of the low-mMV approach is to achieve preservation of the extensor mechanism by means of maximal retention of the VMO muscle bulk attached to the quadriceps tendon, which is crucial for postoperative knee function and recovery. This is accomplished by limiting the sectioning of VMO fibers to just a cuff of 1 cm in width along the inferomedial margin of the VMO, thereby preserving the volume of VMO muscle belly attached to the quadriceps tendon more effectively than traditional mMV techniques.

Our theorized advantages of the low-mMV approach align with core objectives of MIS TKA techniques, including reduced postoperative pain, faster recovery of knee function, and improved patient satisfaction [1]. By maintaining the structural and functional integrity of the VMO, the low-mMV approach aims to optimize extensor mechanism strength and knee stability. Additionally, the reduced soft tissue disruption is expected to decrease inflammatory responses and postoperative stiffness, potentially enabling quicker ambulation and return to daily activities.

In comparison to the traditional mMV approach, which involves a more extensive division of the VMO, the low-mMV approach offers several notable improvements. While some studies have demonstrated the benefits of mMV techniques over medial parapatellar and SV approaches in terms of early postoperative recovery and length of hospital stay, the residual disruption to the VMO in traditional mMV techniques remains a concern [4,8]. By limiting the width of VMO fibers sectioned to 1 cm or less, the low-mMV approach addresses this issue, preserving muscle bulk while ensuring adequate exposure for TKA.

The similarities between the low-mMV and SV approaches necessitate discussion. While the SV technique avoids splitting the VMO entirely, it has been associated with greater difficulty in exposure in certain patient populations, particularly those with obesity or well-developed quadriceps musculature, wherein subluxing the patella and obtaining adequate lateral exposure may be challenging with SV or quadriceps sparing approaches [9]. The low-mMV technique strikes a balance by minimizing muscle disruption while still allowing for adequate visualization and exposure, making it more adaptable across different anatomical variations. The low-mMV approach hence achieves a balance between minimally invasive goals and the practical needs of effective surgical visualization and implant placement. Specific challenges associated with the low-mMV approach include the need for meticulous surgical precision to maintain the integrity of the reduced VMO split and ensure adequate exposure. In cases of patients with morbid obesity or in cases of revision TKA, the utility of this approach may be limited, necessitating alternate more conventional approaches such as medial parapatellar or MV approaches [10].

The low-mMV approach may represent a refinement of minimally invasive TKA; however, further investigation is essential to establish its role within the broader spectrum of MIS techniques. Future studies are required to focus on comparative cohorts that evaluate short-term to long-term outcomes, including functional scores and recovery, and patient-reported satisfaction, in comparison to other minimally invasive and traditional approaches. Additionally, refinement of this technique to incorporate adjunct technologies, such as robotic-assisted surgery or computer navigation, could further enhance the precision and applicability of this technique.

## Summary

The low-mMV approach advances the principles of minimally invasive surgery by maximizing VMO preservation compared to the standard mMV while retaining the flexibility of exposure advantages over the SV approach. This technique represents an incremental but important step forward in the evolution of MIS approaches, with the potential to improve recovery outcomes and patient satisfaction. Further research will determine its place as a standard technique for primary TKA in the context of a rapidly evolving field.

## Conflicts of interest

The authors declare there are no conflicts of interest.

For full disclosure statements refer to https://doi.org/10.1016/j.artd.2025.101686.

## CRediT authorship contribution statement

**Kein Boon Poon:** Writing – review & editing, Supervision, Resources, Project administration, Methodology, Investigation, Formal analysis, Conceptualization. **Justin Zi Xian Chou:** Writing – review & editing, Writing – original draft, Project administration, Formal analysis, Data curation. **Zachariah Gene Wing Ow:** Writing – review & editing, Writing – original draft, Visualization, Validation, Supervision, Software, Project administration, Investigation, Data curation, Conceptualization. **Joel Wei-An Lim:** Writing – review & editing, Visualization, Validation, Supervision, Project administration. **Wei Ming Siow:** Writing – review & editing, Visualization, Validation, Supervision, Project administration, Investigation, Conceptualization.

## Appendix ASupplementary data

Conflict of Interest Statement for Lim

Conflict of Interest Statement for Chou

Conflict of Interest Statement for Poon

Conflict of Interest Statement for Siow

Conflict of Interest Statement for Ow
